# Gut microbial signature in lung cancer patients highlights specific taxa as predictors for durable clinical benefit

**DOI:** 10.1038/s41598-023-29136-4

**Published:** 2023-02-03

**Authors:** Yael Haberman, Iris Kamer, Amnon Amir, Sapir Goldenberg, Gilat Efroni, Inbal Daniel-Meshulam, Anastasiya Lobachov, Sameh Daher, Rotem Hadar, Hadas Gantz-Sorotsky, Damien Urban, Tzipi Braun, Jair Bar

**Affiliations:** 1 Pediatric Gastroenterology, Hepatology and Nutrition Unit, The Edmond and Lily Safra Children’s Hospital, Sheba Medical Center, 52620000 Tel Hashomer, Ramat-Gan, Israel; 2 Department of Pediatrics, Cincinnati Children’s Hospital Medical Center, University of Cincinnati College of Medicine, Cincinnati, OH USA; 3 Institute of Oncology, Sheba Medical Center, 52620000 Tel-HaShomer, Ramat-Gan, Israel; 4 Affiliated with Sackler School of Medicine, Tel-Aviv University, Tel-Aviv, Israel

**Keywords:** Lung cancer, Non-small-cell lung cancer, Microbiome, Cancer

## Abstract

We aimed to determine microbial signature linked with lung cancer (LC) diagnosis and to define taxa linked with durable clinical benefit (DCB) of advanced LC patients. Stool samples for microbial 16S amplicon sequencing and clinical data were collected from 75 LC patients (50 of which were treated with checkpoint inhibitors) and 31 matched healthy volunteers. We compared LC to healthy controls and patients with DCB to those without. LC patients had lower α-diversity and higher between-subject diversity. Random Forests model to differentiate LC cases from controls ROC-AUC was 0.74. Clostridiales, Lachnospiraceae*,* and *Faecalibacterium prausnitzii* taxa abundance was decreased in LC compared to controls. High *Akkermansia muciniphila* correlated with DCB (HR 4.26, 95% CI 1.98–9.16), not only for the immunotherapy-treated patients. In addition, high *Alistipes onderdonkii* (HR 3.08, 95% CI 1.34–7.06) and high *Ruminococcus* (HR 7.76, 95% CI 3.23–18.65) correlated with DCB.Our results support the importance of gut microbiome in LC. We have validated the apparent predictive value of *Akkermansia muciniphila*, and highlighted *Alistipes onderdonkii* and *Ruminococcus* taxa correlation with DCB. Upon additional validations those can be used as biomarkers or as targets for future therapeutic interventions.

## Introduction

Lung cancer (LC) is the number one cause of cancer-related death world-wide. Despite significant advances in the care of non-small cell LC (NSCLC) as well as small cell LC, the majority of patients will die within the first year or two from diagnosis. There is a clear need for earlier diagnosis and for insights into the biology of this disease. Host microbiome composition has been extensively studied for correlation with specific disease states^1^ including LC. One such report regarding gut microbial composition included 30 LC patients^2^—and another study included 18 LC patients^3^. In addition, data from a set of 95 locally advanced NSCLC patients including gut microbiome and urine metabolomics demonstrated high accuracy in differentiating between LC and normal controls^4^. Analysis of 76 early stage NSCLC patients’ gut microbiome identified a signature differentiating between cancer patients and healthy controls with an accuracy of 76.4% in the validation cohort^5^. Salivary microbiome was also found to have a distinct composition in LC patients compared to controls^6^. However, considering the geographic and ethnic variability of gut microbiome composition^7^, such studies require multiple validations.

Fecal microbiome transplant experiments from human cancer patients to mice demonstrated that gut microbiome impacts the response to checkpoint inhibitors (CPIs)^8–10^ and to adoptive cell transfer^11^. The presence or relative abundance of specific microbes such as *Akkermansia muciniphila* or Bifidobacterium^12,13^ correlated with response to anti-PD-1 or anti-PD-L1 checkpoint inhibitors in humans. Interestingly, chemotherapy efficacy has also been linked to microbiome composition^14^, with most data relating to cyclophosphamide and oxaliplatin^15–17^, as well as cisplatin^18^. This association may be related to the role of the immune system in chemotherapy efficacy^19,20^. It can be speculated that targeted agents might also modulate the immune system through specific signaling pathways as well as by exposing immunogenic cancer antigens from dying cells^21^. Two clinical studies of fecal microbial transplant (FMT) combined with CPIs, for CPIs-resistant melanoma and renal cell carcinoma patients demonstrated a proof-of-concept clinical response^22,23^, providing solid evidence for the impact of the gut microbiome on the cancer response to CPIs. In the melanoma study, FMT induced gut gene sets related to antigen presenting cells (APCs) activity, innate immunity, and interleukin-12, as well as increased CD68+ APC cells. Tumor analysis demonstrated enhanced immune-related genes including Interferon-γ signaling and T cell activation, as well as intra-tumoral CD8+ T cell infiltration^22^. The randomized renal cell carcinoma study improved efficacy of the microbe-treated group was accompanied by elevation of circulating inflammatory cytokine blood levels^23^. Considering the expected variations in microbiome composition in different populations^7^, we aimed to profile gut microbiome in LC patients treated in our institution in comparison to age-matched local healthy individuals. The Israeli population is mostly Caucasian, but stemming from various continents and mostly of Jewish ancestry, thus represents a genetically different cohort from western or Asian populations^24^. The most prevalent diet is Mediterranean, which differs from many of previously reported studies of LC patients^25^. We further evaluated in our cohort the correlation between specific bacterial amplicon sequence variants (ASVs) and the long-term disease outcome among the LC patients.

## Methods

### Study design and samples collection

This cohort study was conducted between 2018 and 2021 at the Sheba Medical Center, Institute of Oncology. Consecutive LC patients were recruited, samples and data were collected prospectively. Inclusion criteria were a diagnosis of LC and availability of a stool sample collected prior to any systemic anti-cancer treatment (besides treatments given for earlier stage cancer regarding patients recruited with advanced disease). Healthy volunteers were recruited in parallel; excluded if exposed to antibiotics within six weeks prior to the time of requested sample collection. Fecal samples were obtained using sterile swabs collected from all participants within several hours prior to arrival to the hospital^26,27^. Samples were immediately frozen at − 80 °C upon arrival to the lab until further analyses. Samples from controls and LC patients with or without treatment-related durable clinical benefit (DCB; see below), were handled and processed similarly and included in the same batches. Negative controls (extraction and PCR blanks) were prepared similarly and analyzed together with the rest of the samples. The study was conducted and is currently reported according to the STORMS guidelines^28^.

### Clinical data collection

Clinical and pathological data were collected from medical charts and from questionnaires filled by the participants. Body mass index (BMI) was calculated as weight (kilograms) divided by square height (meters). Performance status was scored by the treating physicians according to the Eastern Cooperative Oncology Group (ECOG-PS) scale (0—no limitations in activity; 4—bedridden). Ethnicity was self-reported, diet or religious group was not collected. Driver mutations were collected when available based on standard-of-care tests performed for advanced NSCLC. Clinical stage was determined based on the American Joint Committee on Cancer (AJCC) staging version 8. Questionnaires regarding antibiotic usage and specific diets were filled out by all study participants at time of sample collection. Response to treatment for LC patients was determined by the treating physicians according to response evaluation criteria for solid tumors (RECIST) version 1.1. Focusing on long-term survival, rate of progression free survival (PFS) at one year was chosen as an endpoint that is more clinically significant than median PFS. Evaluation of disease was performed as part of the standard of care, usually consisting of computerized tomography scans every two to three months.

### 16S rRNA gene amplicon sequencing and bioinformatic analyses

DNA extraction and PCR amplification of the variable region 4 (V4) of the 16S rRNA gene using Illumina adapted universal primers 515F/806R was conducted using the direct PCR protocol [Extract-N-Amp Plant PCR kit (Sigma-Aldrich, Inc.)]^26,27,29^. PCRs were conducted and amplicons were pooled in equimolar concentrations into a composite sample that was size selected (300–500 bp) using agarose gel to reduce non-specific products from host DNA. Sequencing was performed on the Illumina MiSeq platform with the addition of 15% PhiX, generating paired end reads of 175b in length in each direction. Reads were processed using QIIME 2^27,29^ version 2019.7. Quality control was performed by truncating reads after three consecutive Phred scores lower than 20. Reads with ambiguous base calls or shorter than 150 bp after quality truncation were discarded. Amplicon sequence variants (ASVs) detection was done using Deblur^30^, resulting in 149 samples with median of 19,716 reads/sample (mean of 28,911 reads/sample). Taxonomic classification was assigned using a naive Bayes fitted classifier, trained on the August 2013 Greengenes database as the main taxonomy, and additionally on the SILVA release 138 database, for 99% identity^31,32^. For *Akkermansia muciniphila* abundance we calculated the sum of all ASVs matching by taxonomic classification. All samples were rarefied to 3200 reads for α and β-diversity analysis. α rarefaction curves and an additional plot indicating the number of samples left after different rarefaction is shown in Figure S1. The threshold of 3200 reads/samples was chosen to maximize the samples used. Unweighted UniFrac distance was used as a measure of β-diversity, or between sample diversity, and Faith’s phylogenetic diversity was used as a measure of microbial richness, or within sample α-diversity. ASVs heatmaps were generated using Calour version 2019.5.1^33,34^.

Quantifications of microbiome composition variance were calculated using PERMANOVA (Permutational multivariate analysis of variance) with the adonis2 function in the R package Vegan^35^, on the rarefied Unweighted UniFrac distance values. The total variance explained by each variable was calculated while accounting for age and gender in the model. False discovery rates (FDR) were calculated using Benjamini–Hochberg FDR corrections. The random forest analysis was performed in R package randomForest^36^ version 4.6-14 with default parameters. ASVs of 75 LC and 31 age matched control samples were used, including one sample per subject. R AUC package version 0.3.0 was used to calculate the Receiver operating characteristic (ROC) curve and the ROC area under the curve (AUC). MaAsLin2 (Multivariate Association with Linear Models) R package version 1.4.0 was used with default parameters to find ASVs significantly associated with diagnosis or with DCB, after controlling for gender, age, and antibiotic usage, and accounting for samples from the same subject as indicated.

### Statistical analyses

Mann–Whitney U and Yate's Chi squares tests were used to test the statistical differences while using Benjamini Hochberg method to correct for multiple comparisons as needed.

The major outcome measure was 12-months progression free survival, defined as DCB. Kaplan–Meier analyses were done for PFS, evaluating time from treatment initiation till disease progression or death, and censuring patients that were alive with no progression of disease at the last follow up. Hazard ratios for PFS per individualized microbial variation’s Youden most accurate points were computed. Survival/events plots for disease control and related statistics were generated and analyzed using Prism GraphPad version 9.3.1.

### Ethics

All research was performed in accordance with relevant guidelines/regulations. Informed consent was obtained from all participants and/or their legal guardians. The study was performed in accordance with the Declaration of Helsinki and was approved by the Sheba Medical Center ethics committee (approvals #0226-13SMC).

## Results

### Participants and cohort characteristics

LC patients (n = 75), mostly NSCLC, were included in the study. The clinical characteristics are shown in Table 1. All participants (LC patients and controls) were Caucasians. The first sample was collected prior to treatment initiation. A second sample was available from 32 patients, taken on treatment (mean of 40 days on treatment, range 14–111 days). In addition, 31 healthy volunteers with matched median age were recruited as controls. Of these 31, 11 controls have provided a second sample. The total number of samples was 149, originating from 106 subjects. The reasons for failure to collect the second sample were technical (mostly participants’ refusal or neglect).

**Table 1:** Cohort characteristics.

Parameters	Lung cancer	Healthy
N (%)	75 (100)	31 (100)
Age—median (range), years^&^	67 (42–87)	67 (50–81)
Males^&^	41 (55)	15 (48)
Smoking^#^		
Never	13 (17)	20 (64)
Stopped more than 10 years ago	18 (24)	3 (10)
Stopped 1–10 years ago	14 (19)	0 (0)
Current smoker	30 (40)	4 (13)
NA	0 (0)	4 (13)
BMI^&^		
Underweight ≤ 18.5	4 (5.3)	0 (0)
Normal weight = 18.5–24.9	31 (41.3)	8 (26)
Overweight = 25–29.9	28 (37.3)	11 (35)
Obesity = BMI of 30 or greater	12 (16)	3 (10)
NA	0 (0)	9 (29)
Weight loss		
Weight loss more than 5% in 1 year	23 (31)	NA
ECOG PS		NA
0	38 (51)	
1	31 (41)	
2	5 (6.6)	
3	1 (1.3)	
Lung cancer histology		NA
Adenocarcinoma	58 (77.3)	
Squamous cell carcinoma	11 (14.6)	
Large cell/Neuroendocrine	2 (2.6)	
NSCLC—NOS	2 (2.6)	
Small cell carcinoma	2 (2.6)	
Mutations		NA
EGFR	8 (10.6)	
ALK	2 (2.6)	
ROS1	1 (1.3)	
KRAS	14 (18.6)	
Other or NA	24 (32)	
No driver mutations found	26 (34.6)	
Clinical stage		NA
IIB	1 (1.3 )	
III	25 (33.3)	
IV	49 (65.3)	
Durable clinical benefit*		NA
Yes	39 (52)	
No	30 (40)	
NA	6 (8)	
Number of samples	107 (100)	42 (100)
1st sample pre-treatment	75 (70)	31 (74)
2nd sample On treatment	32 (30)	11 (26)
Antibiotic exposure (last 6 weeks)		
Yes	28 (26)	0
No	76 (71)	42 (100)
NA	3 (2.8)	0

All numbers indicate N (%) besides age data. ^&^P-value non-significant (Fisher’s exact test or t-test); ^#^P-value < 0.001 (Fisher’s exact test); both for comparison of lung cancer patients and healthy volunteers. *Durable clinical benefit; 12 months progression free survival. *NA* non-applicable.

LC patients were similar to the healthy control group in terms of age and BMI but had a higher rate of smoking (Table 1). The characteristics of the LC cohort were in general representative of LC patients in terms of age and being mostly males with a high rate of smoking. Most patients had an ECOG PS of 0–1, mostly NSCLC, adenocarcinoma histology and stage IV disease. Driver mutation analysis was available for 68 (90.7%) of the patients. A small subgroup of the NSCLC patients had driver mutations (mostly EGFR). Out of the total 107 samples collected from the LC group, 26% had been collected within six weeks of antibiotics exposure (Dataset S1 includes the biome table with ASVs and taxonomy as well as the indicated metadata).

### Gut microbiome composition of LC patients differs from healthy controls

**Figure 1:**
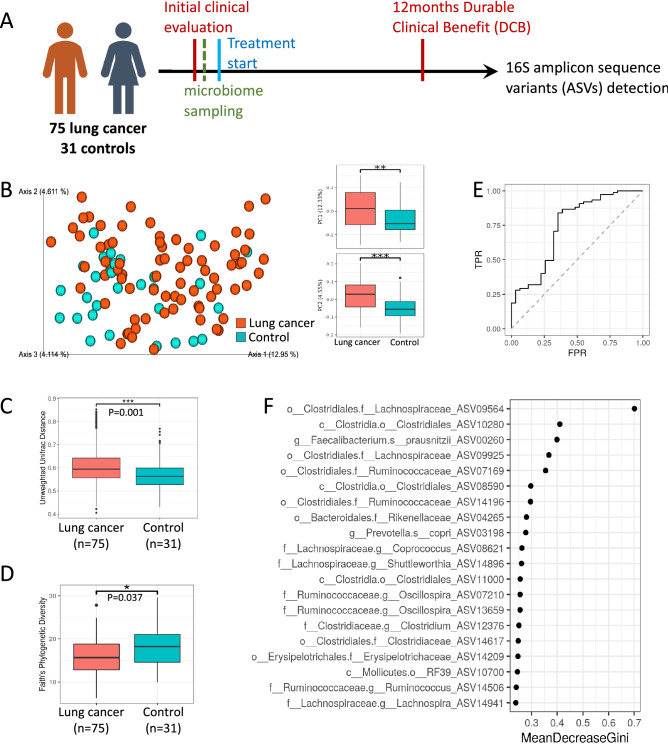
Gut microbial composition is altered in patients with lung cancer (LC). (**A**) Scheme of samples collected design. 106 subjects (75 LC patients and 31 controls) submitted pretreatment fecal samples and had V4 16S amplicon sequencing. (**B**) Unweighted UniFrac PcoA plots colored by disease/healthy (left) and boxplots demonstrating the PC1 and PC2 separation of the groups (right). (**C**) Unweighted UniFrac β-diversity within LC and controls is plotted by diagnosis using only one sample (first sample)/subject (PERMANOVA test, p = 0.001, 999 permutations). (**D**) α-diversity (Faith’s phylogenetic diversity) plotted by diagnosis using only one sample/subject (Mann–Whitney, p = 0.037). (**E**) and (**F**) Random Forest machine learning classification of LC cases from controls using gut microbiome dataset. (**E**) ROC curve of random forest result, differentiating between 75 LC and 31 age matched controls, with an AUC of 0.74. **(**F) Top 20 ASVs used for the random forest result differentiating between 75 LC and 31 age-matched controls (Full list in Dataset S2). Box and whisker plots (**C**–**D**) with central line indicating median, box margins representing upper and lower interquartile region (IQR), and whisker indicates additional 1.5*IQR.

To characterize differences between LC patients and controls we included only one sample per subject and in the case of LC patients this was the sample obtained prior to treatment initiation (75 LC samples and 31 controls, Fig. 1A). To visually explore the variation and similarity between samples’ microbial composition, an unweighted UniFrac based Principal Coordinates Analysis (PCoA) analysis of the cohort was performed (Fig. 1B). Healthy controls predominantly clustered on the right side of the plot and LC samples were mostly on the left as indicated by the respective median PC1 values of 0.093 in controls (IQR: − 0.028 to 0.16) and − 0.028 (IQR: − 0.15 to 0.098) in LC samples (p = 0.001, Mann Whitney U test).

β-diversity was significantly higher among LC patients compared to the diversity among controls, meaning a lower degree of similarity among patients’ gut bacterial composition (Fig. 1C, PERMANOVA P = 0.001 with 999 permutations) To confirm that this effect does not stem from the difference in group size, we redid the analysis using 100 random subsets of 31 LC patients each, to match the number of controls. The resulting p values ranged from 0.001 to 0.004 (data not shown). α-diversity was also assessed, using Faith’s phylogenetic diversity^37^ as a measure of within-sample diversity or microbial richness. A significantly lower within-sample diversity was found in LC cases in comparison to controls (Fig. 1D, Mann Whitney U test P = 0.037).

To evaluate the microbiome as a potential diagnostic tool, we used a supervised learning Random Forests model. To avoid over-fitting bias, only the first sample per patient was used. A receiver operating characteristic (ROC) area under the curve (AUC) of 0.74 was obtained when using 75 LC and 31 control samples (Fig. 1E). The 20 amplicon sequence variants (ASV) taxa with the highest contribution to the classification, as calculated by mean decreased gini^36^ are shown (Fig. 1F; the full list in Dataset S2). The three highest ranking ASV taxa were Clostridiales Lachnospiraceae (ASV09564), Clostridiales (ASV10280), and the short chain fatty acid (SCFA) producer Faecalibacterium prausnitzii (ASV00260) all demonstrating decreased abundance in LC patients in comparison to controls (see Dataset S2 for exact ASV sequences).

**Figure 2:**
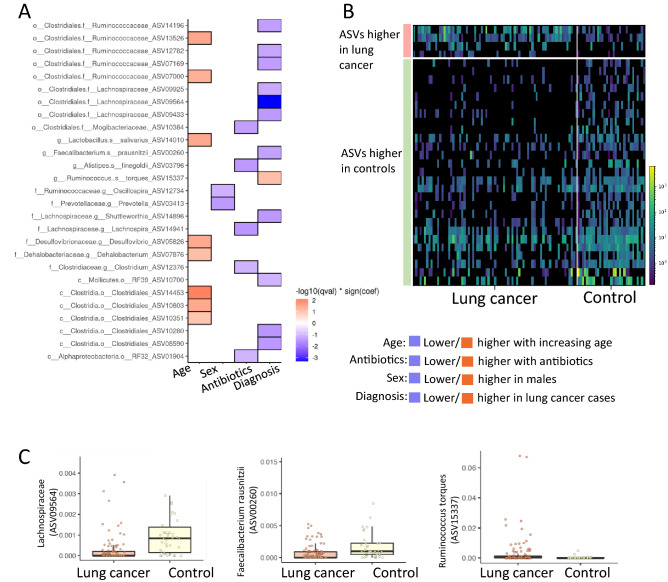
Specific bacterial ASVs linked with LC, after controlling for age, antibiotics, and gender in a multivariate analysis. MaAsLin2 (Multivariate Association with Linear Models) was used to link ASVs to LC in a multivariate model controlling for subject, age, gender, antibiotics. This resulted in 31 bacterial ASVs that showed significant relative differential abundance between LC and controls (FDR correction < 0.25, Dataset S3). (**A)** A heatmap showing the specific bacterial ASV found to be significantly associated with age, gender, antibiotic exposure and LC diagnosis in the model with FDR < 0.1. (**B**) Heatmap of all 31 bacterial ASVs that showed differential abundance between LC and controls (FDR correction < 0.25). Each column represents an individual subject, and each row represents a different bacterial ASV. Color scale indicates the relative frequency out of the normalized reads per sample. ASVs heatmaps were generated using Calour software version 2019.5.1. (**C**) Bar graph highlighting the relative abundance in LC vs. controls of the indicated 3 ASVs linked with LC. Box and whisker plots with central line indicating median, box margins representing upper and lower interquartile region (IQR), and whisker indicates additional 1.5*IQR.

Multivariate analysis was performed by MaAsLin2 (Multivariate Association with Linear Models) linking specific bacterial ASVs with LC diagnosis vs. controls while controlling for gender, age, and antibiotic usage, and accounted for samples from the same subject allowing using the total 149 samples included in the cohort. This analysis showed consistent results with the random forest bacteria ASVs prioritization and resulted in 31 bacterial ASVs linked with LC (p < 0.008 and FDR q ≤ 0.25; Dataset S3), of which 12 ASV were with P < 0.001 and FDR q < 0.1 (Fig. 2A,B). Most ASVs showed reduced abundance in LC patients vs. controls (27/31). Those included ASVs from the Clostridiales order including Lachnospiraceae (the highest ranking; ASV09564, p = 2.32E-07, q = 0.0005) and the above mentioned Faecalibacterium prausnitzii (ASV00260, p = 0.0004, q = 0.045) (Fig. 2B,C and Dataset S3). In contrast, Ruminococcus torques (ASV15337, p = 0.0008, q = 0.075) showed higher relative abundance in subjects with LC (Fig. 2A–C). This analysis also highlighted 21 ASVs associated with age, 14 ASVs with gender, and 13 ASVs with antibiotics use (Dataset S3).

### Higher *Akkermansia muciniphila* abundance is associated with DCB

**Figure 3:**
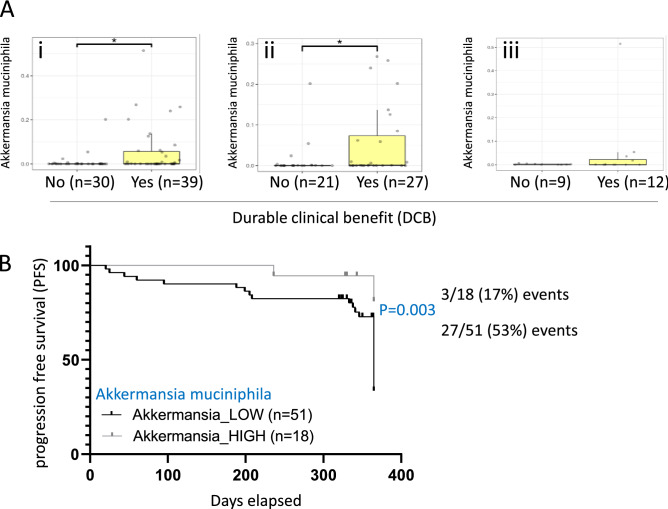
Higher *Akkermansia muciniphila* abundance is associated with durable clinical benefit (DCB). (**A**) Bar graph indicates pretreatment *Akkermansia muciniphila* abundance stratified by patients with and without DCB in all patients for which this data was available (n = 69; i), only in the group treated with immunotherapy (n = 48; ii), or the sub-group of LC patients not treated by CPIs (n = 21, iii). * Indicates p < 0.05 using t-test. (**B**) Patients with and without DCB for which this data was available (n = 69) were included. Youden point of 0.0074 was calculated to discriminate between those with and without DCB. Based on that value 69 patients were stratified to *Akkermansia muciniphila* low or high (above 0.0074) for Kaplan–Meier PFS analyses [Log-rank (Mantel-Cox) test P value = 0.003, and Hazard Ratio 4.26 (95% CI of ratio 1.98–9.16)].

Focusing on LC patients, we were interested to find a potential correlation between specific components of the gut microbiome and long-term disease control. One-year progression free survival (PFS) was chosen as the relevant outcome measure, defined as DCB. The distribution of patients with or without DCB in our study between the treatment groups as well as antibiotic exposure is presented in supplementary Table S1, full data is in Dataset S1. Since *Akkermansia muciniphila* was previously shown to be linked with outcome, we tested if *Akkermansia muciniphila* abundance is linked with DCB also in our cohort. Importantly, higher *Akkermansia muciniphila* abundance was associated with DCB as demonstrated in Fig. 3A, for all LC patients (i) as well as only regarding CPIs-treated patients (ii). In the sub-group of LC patients not treated by CPIs (n = 21) there was a trend toward better response by Akkermansia abundance (Fig. 3Aiii). We could not find evidence for the recently reported bi-phasic impact of *Akkermancia muciniphila* abundance on outcome of CPIs-treated patients^38^. We calculated a Youden point of 0.0074 to best discriminate between DCB+ and DCB− patients and accordingly stratified the 69 patients with available data to *Akkermansia muciniphila* low or high, comparing them by Kaplan–Meier PFS analysis. The patients with high *Akkermansia muciniphila* had significantly higher odds for DCB [Log-rank (Mantel-Cox) test P value = 0.003, and Hazard Ratio 4.26 (95% CI of ratio 1.98–9.16)] (Fig. 3B).

### Specific ASVs linked with DCB after controlling for factors linked with microbial variance

**Figure 4:**
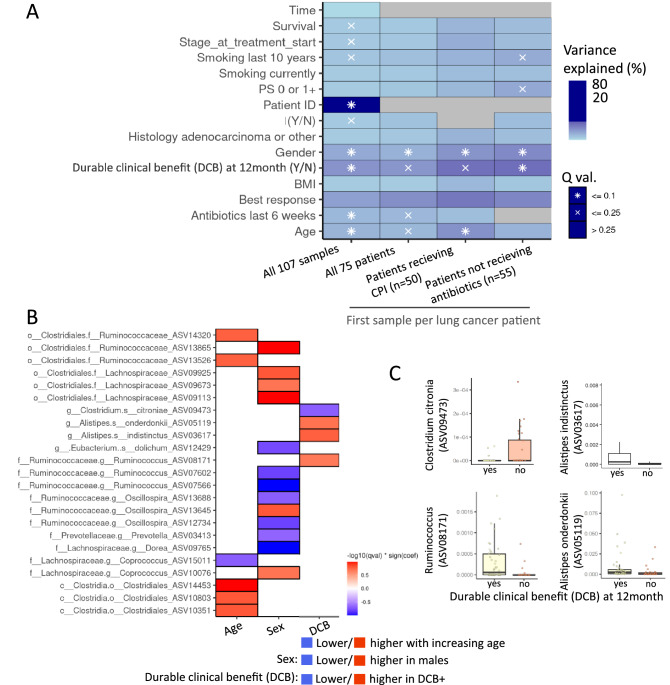
Specific ASVs linked with DCB in a multivariate model. (**A**) PERMANOVA analysis of factors explaining microbiome variance. Inter-individual sample variation explains most of the microbiome composition variation (left column, all 107 samples). When considering only one sample per patient in all the LC group (n = 75), those receiving immunotherapy, and those not receiving antibiotics, gender and DCB remain significant. Variance is estimated for each feature independently, while accounting for age, gender besides for when considering age or gender (see “Methods”) (Dataset S4). Included n for each subgroup is shown in brackets. (**B**) MaAsLin2 (multivariate association with linear models) was used to link ASVs to age, gender, and to DCB (controlling for subject, age, gender) in a multivariate model (Dataset S5). This resulted in 4 bacterial ASVs that showed significant relative differential abundance between DCB+ and DCB− patients. (Results after FDR correction < 0.25, p < 0.005). (**C**) Bar plot of those 4 ASVs taxa are shown.

To quantify the contribution of different factors affecting the gut microbial composition, we used a PERMANOVA test (Fig. 4; Dataset S4). PERMANOVA was applied after controlling for age and gender for each parameter, except when evaluating for the role of age and gender (in which cases we controlled for either gender or age). Figure 4A demonstrates the factors significantly linked to microbial variance as well as the level of explained variance. As expected, when including all samples of the LC group, inter-patient variance was the predominant factor. To control for the contribution of the subject, we limited our further analyses and included only one sample per subject. Importantly, when only the first sample of each patient was included, as well as when focusing on patients that have received immunotherapy and on those that did not receive antibiotics, gender and DCB remain significantly associated with microbial composition while most other factors do not. Patients with and without DCB did not show differences in within-sample α-diversity (data not shown). However, multivariate analysis (using MaAsLin2) linking specific bacterial ASVs with DCB while controlling for gender, age, and accounting for samples from the same subject identified four ASVs that remain significantly correlated with DCB (after controlling also for age and gender, Fig. 4B,C, Dataset S5). Those included higher *Alistipes indistinctus* (ASV03617), Ruminococcus (ASV08171) and *Alistipes onderdonkii* (ASV05119) in those with DCB and higher *Clostridium citroniae* (ASV09473) in those without DCB.

**Figure 5:**
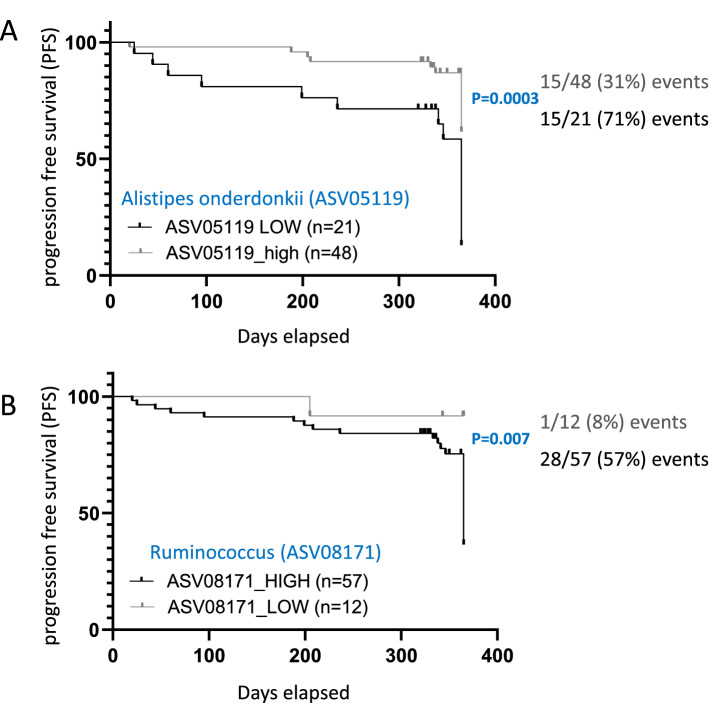
Higher *Alistipes onderdonkii* (ASV05119) and Ruminococcus (ASV08171) abundance is associated with durable clinical benefit (DCB). 69 patients with available data were included. (**A**) Regarding *Alistipes onderdonkii* (ASV05119) data, a Youden point of 0.00018 was calculated to discriminate between DCB+ and DCB− patients; based on that value 69 patients were stratified to low or high (above 0.00018) for a Kaplan–Meier DCB analysis [Log-rank (Mantel–Cox) test P value = 0.0003, and Hazard Ratio 3.08 (95% CI of ratio: 1.34–7.06)]. (**B**) Regarding Ruminococcus (ASV08171), a Youden point of 0.0004 was calculated to discriminate between DCB+ and DCB− patients; based on that value 69 patients were stratified to low or high (above 0.0004) for a Kaplan–Meier DCB analysis [Log-rank (Mantel–Cox) test P value = 0.007, and Hazard Ratio 7.76 (95% CI of ratio: 3.23–18.65)].

We then calculated a Youden point that best discriminates between DCB+ and DCB− patients for those four bacterial ASVs taxa and accordingly stratified the 69 patients with available data to low or high abundance of each indicated ASV for Kaplan–Meier PFS comparison. Two of these four, *Alistipes onderdonkii* (ASV05119) Ruminococcus (ASV08171) (Fig. 5) showed significant results in Kaplan–Meier PFS, supporting the importance of these taxa. For *Alistipes onderdonkii*, Youden point of 0.00018 was calculated to discriminate between those with and without progression free survival (PFS) at 12 months. Patients with high *Alistipes onderdonkii* had significantly higher odds for DCB [Log-rank (Mantel-Cox) test P value = 0.0003, and Hazard Ratio 3.08 (95% CI of ratio 1.34–7.06)]. Youden point of 0.0004 was used to stratify Ruminococcus abundance. Patients with high Ruminococcus had significantly higher odds for DCB [Log-rank (Mantel-Cox) test P value = 0.007, and Hazard Ratio 7.76 (95% CI of ratio 3.23–18.65)]. The other two ASVs taxa did not demonstrate significant difference when comparing the high and low groups in the Kaplan–Meier PFS analyses.

## Discussion

We report here the results of a cohort of LC patients with detailed clinical and gut microbiome analysis including 75 highly phenotyped LC patients and 31 controls. We have captured significant differences between LC patients and controls at the community and taxonomy levels (α- and β-diversity). We have identified lower within-sample α-diversity in LC samples vs. controls, as reported previously^2^, and reduction of ASVs from the Clostridiales order including *Lachnospiraceae* and the short-chain fatty acids producer *Faecalibacterium prausnitzii*. Importantly, we demonstrate a significant correlation within the LC patients group between the gut microbiome composition and patients’ outcome. Interestingly, this effect was not limited to the CPIs-treated group; out of 69 patients for which outcome data was available, 48 had received CPIs while 21 did not. In fact, patients’ outcome (DCB) was one of the most significantly correlated factors with gut microbial composition within the LC population as shown in the PERMANOVA analyses, explaining 3.4–5.2% of the microbial variation. This level is higher than the variance explained by antibiotics treatment prior to sampling. Our data demonstrates associations between the gut microbiome and LC as well as its potential use as a biomarker for response to treatments, or alternatively as a prognostic marker. Importantly, our data supports the gut microbiome as a possible therapeutic target that can be manipulated specifically to improve outcome.

Previous studies regarding specific bacteria association with LC diagnosis identified several relevant species. *Clostridiales Lachnospiraceae* was reported to be decreased in gut microbiome of LC cases (n = 30) compared to controls (n = 16)^2^ similar to our results. This microbe was also found lower in salivary microbiome of LC cases (n = 20) versus healthy individuals (n = 10)^6^. *Faecalibacterium prausnitzii* was also reported as lower in the gut microbiome of LC (n = 34, 30)^5,14^ as was seen here. In contrast, *Ruminococcus torques* which was higher in LC cases in our study has not been reported so far to be related to LC diagnosis. Other microbial correlations with LC reported here are novel. This may be explained by geographical, diet and ethnic variation and emphasize the need for additional studies including diverse populations.

The benefit from CPIs in advanced NSCLC has been previously found to correlate with certain bacterial species in the gut microbiome, some of them also identified in our current study, most prominent of those being *Akkermansia muciniphila*. Interestingly, in multivariate analysis including all identified microbes from our study, microbes other than *Akkermansia muciniphila* correlated to a higher extent with DCB. The cohort-dependent correlation of specific gut microbes with CPIs-benefit was recently highlighted in a multi-cohort melanoma study^39^. Examples for microbes found to strongly and positively correlate with DCB in our study include *Ruminococcaceae*, which was also reported to be enriched in the gut of advanced Japanese NSCLC patients (n = 70) surviving longer than 12 months^40^. *Ruminococcaceae* species were also common in melanoma patients (n = 38, stage III) gut samples among responders to neoadjuvant anti-PD1 and anti-CTLA4 combination^41^. *Alistipes indistinctus*, another prominent microbe in our results was more abundant in stool samples of responders vs. non-responders in a set of 56 NSCLC patients^8^, similar to our results. In a mouse model, this bacterium restored responsiveness to CPIs when given to non-responders^8^. Another study conducted in China with patients with advanced NSCLC (n = 37) undergoing anti-PD-1 immunotherapy, identified enrichment of a different species of Alistipes, *Alistipes putredinis*, in addition to *Bifidobacterium longum*, and *Prevotella copri* in patients responding to this treatment^42^. These bacteria were not found to be correlated with DCB in our study, again highlighting the importance of geographic and ethnic variability in gut microbiome and its role in cancer. It should be noted also that response to treatment was the endpoint of some of the studies^8,42^, which is not equivalent to DCB, the endpoint we chose to focus on, considering DCB to be clinically more important. Along those lines, higher *Clostridium citronia* was found in our data to be linked with poorer prognosis, which was not previously reported. Interestingly, *Alistipes onderdonkii*, significantly correlated with DCB in our dataset, was found to be attenuated in the gut of a mouse model of pancreatic cancer, and the supernatant of this microbe suppressed the proliferation of the pancreatic cancer cells^43^.

The involved mechanisms linking treatment efficacy and gut microbiome are not clear. In general, the gut microbiome thrives in close association with the local gut immune system, and through local interactions or systemically circulating derivates, can impact the host immune system in multiple manners^44^. A few gut microbes induce antigen-specific T-cell responses. One of those is *Akkermansia muciniphila*, where specific T cell expansion can occur, dependent on the context of a conventional microbiota^45^. *Akkermansia muciniphila* has also been reported to present a membrane protein (Amuc-1100) that can activate immune Toll-like receptor 2^46^. Additional suggested mechanisms for microbiome influence on the immune system and on the efficacy of anti-cancer drugs include impact on dendritic cell activity in the gut lamina propria^47^ and in tumor-draining lymph nodes^12^, systemic activity of metabolic bacterial products^47^, molecular mimicry between specific bacteria and cancer antigens^48,49^, or migration of bacteria from the gut to lymphatic organs or to the tumor itself^16,47,50^. Bacterial-produced metabolites such as short-chain fatty acids (SCFA; mostly acetate (C2), propionate (C3) and butyrate (C4)) might be specific mediators of the effect of some bacteria and immune cells^51^, shown to inhibit the effect of anti-CTLA-4 in a melanoma model. The correlation of microbiome components and response to non-immunotherapy drugs suggested by our study may occur also through the impact of the gut microbiome on the immune system. It can be speculated that targeted agents might also modulate the immune system through specific signaling pathways as well as by exposing immunogenic cancer antigens from dying cells^21^. The correlation of *Akkermansia muciniphila* with DCB across various cohorts supports the importance of this microbe for cancer control. *Akkermansia muciniphila* should be further investigated as a potential therapeutic tool for LC patients, by gut microbial transplant procedures or by use of specific components of this taxa.

Our study has several strengths and some limitations. The use of a relatively large, prospective and longitudinally cohort with detailed clinical and microbial characterization has enabled identifying microbial factors linked to diagnosis and outcome of LC patients. Limitations of this study include the heterogeneity of the cohort and the mixture of administered treatments. However, the reproduction of previously reported results in this cohort despite these limitations lends further credibility to our data. Another limitation is the use of 16S rRNA amplicon sequencing (rather than shotgun data) which limits our ability to identify associated bacterial metabolic pathways and functions, and to get resolution at the species level^52^. The size of our study group is one of the largest among the reported studies; however, larger studies in diverse populations are required to provide robust data about some of our findings that did not reach strong statistical value. Another potential limitation is that only in the control arm antibiotic exposure within six weeks prior to the time of sample collection was an exclusion criteria. Prolonged longitudinal sampling of the gut microbiome of our patients, correlating the persistence of specific taxa of interest with DCB is lacking. A recognized limitation of microbiome studies is the multiplicity of potential confounding factors. For example, smoking could impact microbiome composition^53^, a factor we could not control for in the comparison between LC and healthy volunteers. In addition, various medications could impact the microbiome^54^, another example of a feature we did not control for. Nevertheless, multivariate analysis of our data retains the significant correlation between certain microbes and LC diagnosis as well as the outcome of LC patients. The molecular mechanisms underlying these correlations require further studies.

To conclude, we have profiled the gut microbiome and identified specific microbial taxa differentiating LC patients from age and gender-matched healthy individuals. Within LC patients we identified specific bacterial amplicon sequence variants (ASVs) linked with the long-term disease outcome, including *Alistipes onderdonkii* (ASV05119) and *Ruminococcus* (ASV08171). Our data and other reports^39^ suggest specific microbes’ roles in cancer might be geographically, diet and/or ethnically-specific. Nevertheless, we have validated in our cohort, which is different geographically and ethnically from previous cohorts and includes patients treated with and without CPIs, that *Akkermansia muciniphila* correlates with better outcome^38^. After additional validations, these microbes can be used as biomarkers for treatment response or possibly of overall better outcome regardless of the type of treatment. Additionally, those can potentially be used as targets for therapeutic manipulations.

## Supplementary Information

Supplementary Information 1.

Supplementary Information 2.

Supplementary Information 3.

Supplementary Information 4.

Supplementary Information 5.

Supplementary Table 1.

Supplementary Figure 1.

## Data Availability

The study datasets were deposited at the National Center for Biotechnology Information as BioProject PRJNA805069. Reviewers’ link: https://dataview.ncbi.nlm.nih.gov/object/PRJNA805069?reviewer=1fddkcjvbne55q90n7c8830mmp.
