# Supplementary material for: Gut microbial signature in lung cancer patients highlights specific taxa as predictors for durable clinical benefit
Source: Sci Rep. 2023 Feb 3;13:2007. doi: 10.1038/s41598-023-29136-4 (PMC9898251; doi:10.1038/s41598-023-29136-4)
Supplement: Supplementary file 6 — Supplementary Table 1. [file 41598_2023_29136_MOESM6_ESM.docx]

**Table S1: Treatments and antibiotics use**

|  | **Durable disease control –**  **N (% of each treatment group) *** | | | |
| --- | --- | --- | --- | --- |
| Treatment groups | **Yes** | **No** | **NA** | **Total** |
| CPI | 3 (37.5) | 4 (50) | 1 (12.5) | 8 |
| Chemo- CPI | 12 (46) | 13 (50) | 1 (3.8) | 26 |
| Chemo-Rad- CPI | 12 (75) | 4 (25) | 0 (0) | 16 |
| Other | 12 (48) | 9 (36) | 4 (16) | 25 |
|  | **Antibiotics exposure –**  **N (% of each treatment group) #** | | | |
| Treatment groups | **Yes** | **No** | **NA** | **Total** |
| CPI | 4 (25) | 12 (75) | 0 (0) | 16 |
| Chemo- CPI | 12 (26.6) | 33 (73.3) | 0 (0) | 45 |
| Chemo-Rad- CPI | 4 (19) | 16 (76.2) | 1 (4.8) | 21 |
| Other | 8 (32) | 15 (0.6) | 2 (8) | 25 |

*12 months progression free survival. #Within the prior six weeks.

CPI: checkpoint inhibitors. Chemo: chemotherapy. Rad: radiotherapy. NA: non-applicable.
