# Supplementary material for: Gut microbial signature in lung cancer patients highlights specific taxa as predictors for durable clinical benefit
Source: Sci Rep. 2023 Feb 3;13:2007. doi: 10.1038/s41598-023-29136-4 (PMC9898251; doi:10.1038/s41598-023-29136-4)
Supplement: Supplementary file 7 — Supplementary Figure 1. [file 41598_2023_29136_MOESM7_ESM.pdf]

Supplamentary figure

A

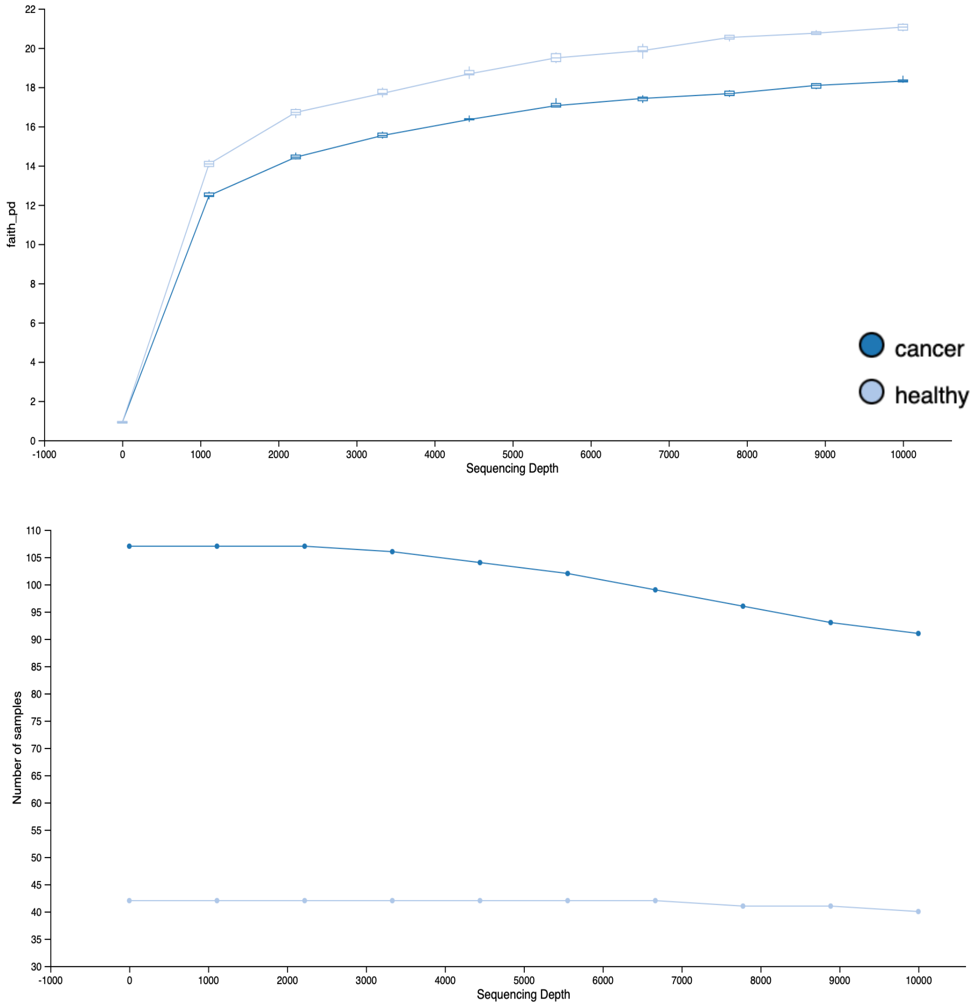

B

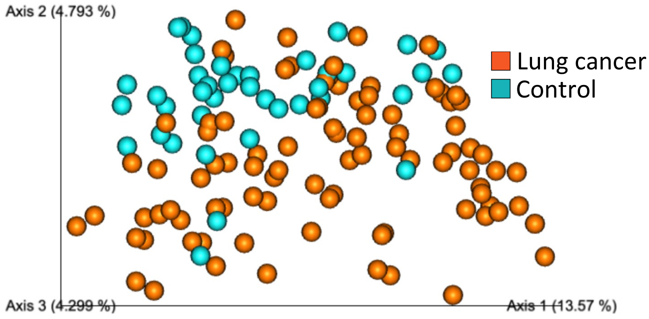

Figure S1| **Alpha rarefaction and read depth. A.** Alpha rarefaction curves (upper panel) and a plot indicating the number of samples left after different rarefaction (lower panel). **B**. PCoA plot using rarefaction of 10000 reads.
